# Supplementary material for: Estimating firms' emissions from asset level data helps revealing (mis)alignment to net zero targets
Source: Nat Commun. 2026 Mar 7;17:3640. doi: 10.1038/s41467-026-70481-5 (PMC13096287; doi:10.1038/s41467-026-70481-5)
Supplement: Supplementary file 1 — Supplementary Information [file 41467_2026_70481_MOESM1_ESM.pdf]

Estimating firms' emissions from asset level data helps revealing (mis)alignment to net zero targets

Hamada Saleh[1], Stefano Battiston[2,3], Irene Monasterolo[4,5,6], Thibaud Barreau[1], Peter Tankov[7,1*]

[1]Institut Louis Bachelier, Paris, France.
[2]University of Zurich, Zurich, Switzerland.
[3]University of Venice, Venice, Italy.
[4]University of Utrecht, Utrecht, Netherlands.
[5]CEPR, Paris, France.
[6]WU Wien, Vienna, Austria.
[7]CREST, ENSAE, Institut Polytechnique de Paris, Palaiseau, France.

*Corresponding author(s). E-mail(s): peter.tankov@ensae.fr;

1 Alignment of iron and steel sector with APS and STEPS scenarios

In the main body of the paper we have compared the projected bottom-up emissions of the steel sector to the reference emission trajectory for this sector from the IEA Net Zero 2050 scenario and found a considerable mismatch. We now perform the same comparison taking as reference trajectories for emissions and for global steel production those of APS and STEPS scenarios. The results are shown in Supplementary Figure 1 (APS scenario) and Supplementary Figure 2 (STEPS scenario).

Under global steel production from APS scenario, in the most optimistic case, assuming that the electricity sector decarbonizes at the net zero rate and all best available technologies for BF-BOF production route decarbonization are implemented, bottom-up steel sector emission trajectory undershoots the reference emission trajectory of the APS scenario by 3%, while under the least optimistic assumptions, there is a 9% overshoot.

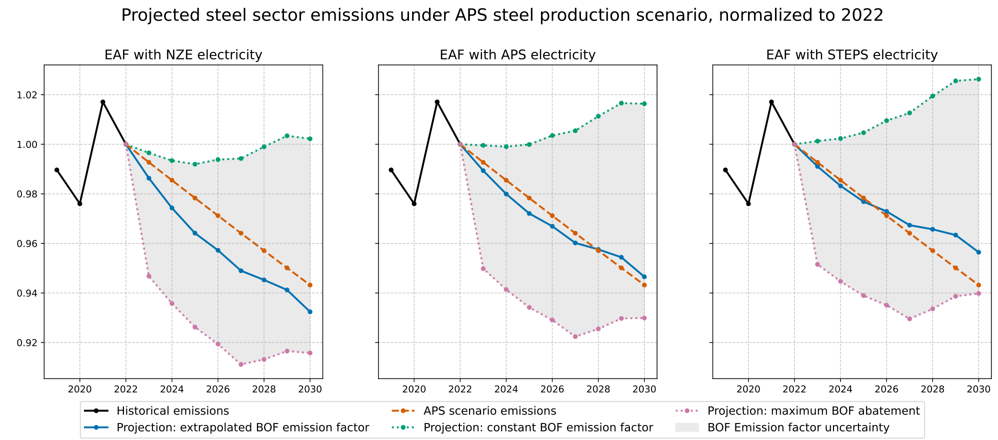

Supplementary Figure 1: Projected bottom-up emissions of the Iron & steel sector under different assumptions on the future evolution of emission factors, compared to APS scenario emissions, normalized to 1 in 2022. In the left chart, the EAF emission factor is computed assuming that the carbon intensity of electricity production follows the NZE scenario, in the middle chart the APS scenario, and in the right chart the STEPS scenario. The upper dotted curve is computed assuming that the emission factor of BF-BOF technology is constant, the middle solid uses historical extrapolation from past 20 years, and the lower dotted curve assumes full implementation of best available technologies for the decarbonization of BF-BOF process (see Methods section for details).

Under global steel production from STEPS scenario, in the most optimistic case, bottom-up steel sector emission trajectory undershoots the reference emission trajectory of the STEPS scenario by 9%, while under the least optimistic assumptions, there is a 3% overshoot.

2 Sources of uncertainty in the asset level emission projection methodology

In this section, we examine various sources of uncertainty of our methodology.

Choice of scenario for projecting future production and electricity carbon intensity and for alignment assessment

Our study is based on the IEA World Energy Outlook (WEO) 2023 scenarios. To illustrate the amplitude of uncertainty related to scenario choice, we compare the relevant variables from these scenarios with the corresponding variables from another widely used scenario source, namely the NGFS scenario database.

Release IV of the NGFS scenario database contains 7 transition scenarios: Net Zero 2050, Low Demand, Below 2° C, NDCs, Current Policies, Delayed Transition and Fragmented World. The Net Zero 2050 NGFS scenario can be naturally identified

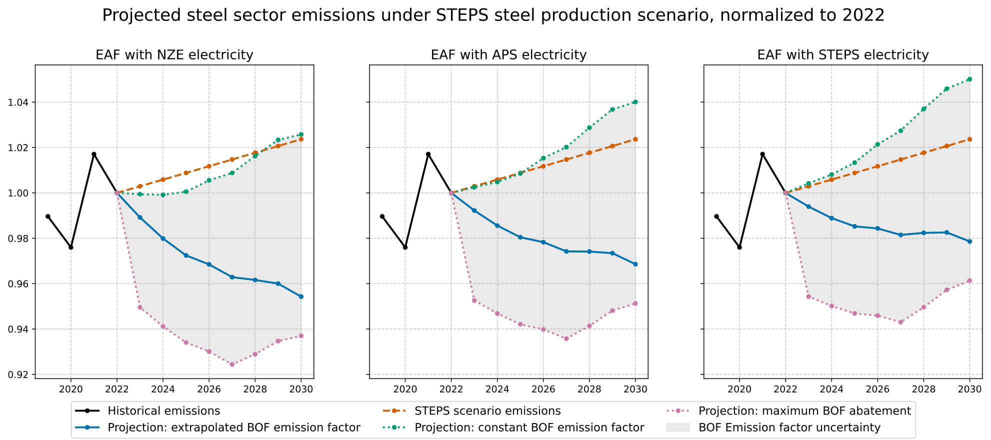

Supplementary Figure 2: **Projected bottom-up emissions of the Iron & steel sector under different assumptions on the future evolution of emission factors, compared to STEPS scenario emissions, normalized to 1 in 2022.** In the left chart, the EAF emission factor is computed assuming that the carbon intensity of electricity production follows the NZE scenario, in the middle chart the APS scenario, and in the right chart the STEPS scenario. The upper dotted curve is computed assuming that the emission factor of BF-BOF technology is constant, the middle solid uses historical extrapolation from past 20 years, and the lower dotted curve assumes full implementation of best available technologies for the decarbonization of BF-BOF process (see Methods section for details).

with the NZE2050 scenario from WEO. The APS scenario of WEO corresponds to 2100 warming level of $1.7°C$, we therefore identify it with the NGFS Below 2° C which corresponds to the same projected warming level. Finally, the STEPS scenario of WEO corresponds to 2100 warming level of $2.4°C$ and is therefore identified with the NDCs NGFS scenario. Supplementary Table 1 presents the correspondence between NGFS and IEA scenarios. NGFS scenarios contain data from several different models; for comparison we use REMIND-MAgPIE and MESSAGEx-GLOBIOM.

Supplementary Figure 3 plots the projections of carbon intensity of electricity production in the three scenarios we study, for the two NGFS models and for the IEA data. We see that the two alternative NGFS projections and the IEA projections are quite close to each other; moreover, for Net Zero and the Announced Pledges scenario, the IEA projection is slightly higher than the two NGFS projections; thus, our estimates, based on the IEA projections, are conservative in this case. In the Stated Policies scenario, the IEA projection is between the two NGFS projections. Supplementary Figure 4 plots the IEA projection and the two NGFS projections of the steel production in the three scenarios we study. Here, the IEA projection is between the two NGFS projections at all times, but the two NGFS projections are very different from each other, in particular, the projection obtained using REMIND-MAgPIE model has a strong decreasing trend in the Net Zero and the anounced pledges scenario, while the projection of MESSAGEix-GLOBIOM model has an increasing trend in all

NGFS scenarios	2100 warming level	WEO 2023 scenarios	2100 warming level
Net Zero 2050	1.4°C	NZE2050	1.4°C
Below 2° C	1.7°C	APS	1.7°C
NDCs	2.4°C	STEPS	2.4°C
Low Demand	1.4°C		
Current Policies	2.9°C		
Delayed Transition	1.7°C		
Fragmented World	2.3°C		

Supplementary Table 1: End-of-century warming levels for NGFS Release IV and IEA WEO 2023 scenarios. Sources: iea.org/reports/world-energy-outlook-2023/ and ngfs.net/en/ngfs-climate-scenarios-phase-iv-november-2023.

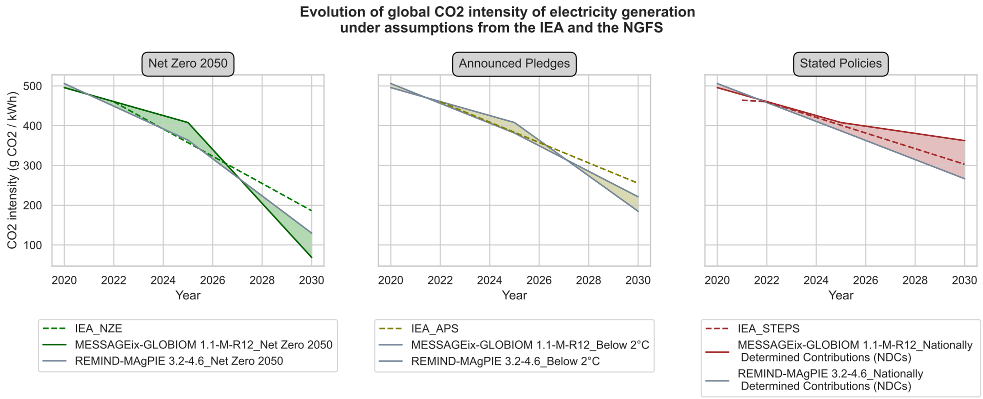

Supplementary Figure 3: **Comparison of CO2 intensity trajectories of electricity generation under assumptions from the IEA and the NGFS.** Each trajectory is based on the pairing of a climate model (GCM) and a climate scenario. NGFS trajectories are represented with filled lines and IEA trajectories are represented with dashed lines. While values are directly accessible from the IEA, we reconstruct CO2 intensity from NGFS data using the variables 'Emissions|CO2|Energy|Supply|Electricity' and 'Production|Steel'.

scenarios. Moreover, the two models give different steel production values even in the historical period. We conclude that the NGFS projections are less reliable in this case and base our estimates on the IEA projection.

Data on future openings and closures of assets

An important ingredient of our method is the information of future opening and closure dates of assets, which may not always be readily available. We believe that while future openings of electric and DRI plants may be rather well represented in the database, the information about future closures of blast furnace plants, although present in the database, may not be complete. To mitigate this issue, as explained in the methodology section, we add artificial closure dates of the oldest blast furnace plants to the database

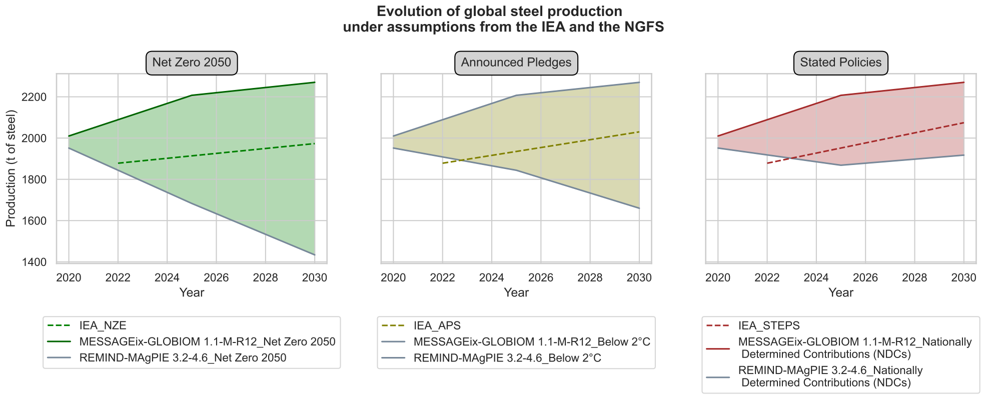

Supplementary Figure 4: **Comparison of global steel production trajectories under assumptions from the IEA and the NGFS.** Each trajectory is based on the pairing of a climate model (GCM) and a climate scenario. NGFS trajectories are represented with filled lines and IEA trajectories are represented with dashed lines. We notice that IEA trajectories fall well within the bounds of NGFS trajectories.

to meet the scenario-based global capacity constraint. Supplementary Table 2 gives the number of plants removed from the database in each year and for each scenario. The total number of plants removed in this way ranges from zero in the STEPS scenario to 29 in the NZE 2050 scenario, and remains small compared to the total number of plants in the database (950). We conclude that although this data incompleteness issue may create an upward bias in our results, this bias remains limited. Also, the lower bound on projected bottom-up emissions (see Figure 2 of the main document) which is computed under the assumption that only the least carbon intensive plants are used, is not sensitive to this bias. Since our main qualitative conclusions remain valid for this lower bound, we conclude that they are robust with respect to this source of uncertainty.

**Scenario**	**2023**	**2024**	**2025**	**2026**	**2027**	**2028**	**2029**	**2030**
**NZE**	0	0	11	2	2	2	2	10
**APS**	0	0	4	2	0	0	0	7
**STEPS**	0	0	0	0	0	0	0	0

Supplementary Table 2: **Number of plants artificially removed from the database based on global capacity constraint.** We assume global capacity evolves at the same rate as production in NZE, APS and STEPS scenarios. For each year between 2023 and 2030, we generate a list of operating plants, which is solely based on available opening and closure dates. Capacity aggregated at the plant level may exceed the previously calculated global capacity level, which is scenario-dependent. In such a case, we remove the oldest blast furnace (BF) plants, until the constraint is met.

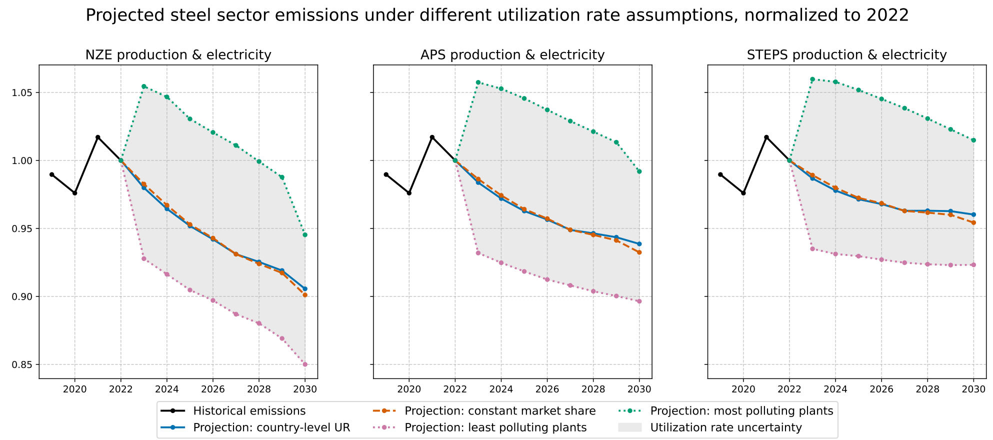

Supplementary Figure 5: Projected steel sector emissions under different assumptions on the future utilization rates of steel plants. The three graphs correspond to three different scenarios of global steel production and carbon intensity of electricity production. The BF-BOF emission factor is obtained by historical extrapolation from past 20 years. The dotted curve computes the utilization rate of each plant using the constant market share method, the solid curve uses the country-level utilization rate method, the lower dotted curve uses the least polluting plants in each country to match the projected country-level production and the upper dotted curve uses the most polluting plants.

Assumptions on future utilization rates of assets

Our results are also sensitive to the assumptions about the future utilization rates of steel-making plants. Since the global steel production is based on scenarios, the assumptions about the utilization rate impact the distribution of the global production effort among different plants. As explained in the methodology section, we explore this source of uncertainty in two ways: (a) by considering two alternative projection methods, the first one based on country-level utilization rate and the second one based on market shares of companies; and (b) by exploring two extreme cases, where only the most polluting or only the least polluting plants are used. To complement the graph illustrating the impact of utilization rate uncertainty under the net zero scenario of electricity carbon intensity and steel production, Supplementary Figure 5 shows the impact of utilization rate uncertainty under all three scenarios of IEA WEO 2023. We see that the utilization rate uncertainty can modify the 2030 emission estimate by at most 5% which does not qualitatively change the conclusions of our study.

Assumptions on future carbon intensity of production

Finally, a major source of uncertainty in our estimates is related to the future evolution of the carbon intensity of steel production technologies. This uncertainty is analyzed in detail in the main paper and the corresponding bounds are shown in the graphs.

3 Cross-sector applicability

In this section we discuss the applicability of our methodology to other industrial sectors. Our methodology requires identifying the main decarbonization pathways for the specific sector, and assessing the future impact of these decarbonization pathways on the sector emissions, using the following sector-dependent data:

- Plant-level data: capacity, production technology and production volume of each plant; projected opening dates of future plants and closing dates of existing ones.
- Technology data: present and future emission factors for each technology.

We discuss decarbonization pathways, the availability of these data and specific challenges for applying our methodology, focusing on cement, aluminum, pulp and paper and power sectors. Specific references to plant level data sets and technology-related information for these sectors are provided in Supplementary Table 3.

Cement sector

Cement production accounts for approximately 8% of human-made carbon dioxide emissions. The production of clinker (main ingredient of ordinary Portland cement, OPC) by limestone calcination makes up approximately 50% of carbon emissions with further 40% coming from energy consumption and 10% from mining and transportation. The main decarbonization pathways are therefore (a) substituting clinker with other materials (so called supplementary cementous materials (SCM), such as fly ash) which produce less CO2 emissions; (b) fuel decarbonization, switching from fossil fuels to low-carbon alternatives for kiln heating; and (c) carbon capture and storage [1].

The specific challenges for the cement sector are related to weaker data availability than for the steel sector. Plant-level transparency is limited, and emission factors vary with clinker ratio, fuel mix, and kiln type, with inconsistent reporting across countries.

Aluminum sector

The aluminum sector is responsible for about 2-3% of global GHG emissions. The main decarbonization pathways for the aluminum sector [5] are (a) Electricity decarbonization. Primary aluminum production is an electrical energy-intensive process, accounting for 60% of total emissions of this sector; (b) Direct emissions reduction. Direct emissions from fuel combustion make up 15% of sector emissions and can be reduced through electrification, use of green hydrogen, or CCUS technologies; (c) recycling and resource efficiency.

The aluminum industry has data gaps compared to steel. While the International Aluminium Institute (IAI) publishes global and regional energy and CO_2 intensity figures, plant-level data are scarce, and differences in electricity sources (coal, hydro, renewables) make country comparisons difficult.

Pulp and paper sector

The pulp and paper sector was responsible for just under 2% of all emissions from industry in 2022. Paper production is very heat-intensive due to large amounts of water to be evaporated and the overwhelming majority of GHG from the pulp and

Technology	Plant-level data	Technology data
**Cement**	Global Cement and Concrete Tracker (globalenergymonitor.org/projects/global-cement-and-concrete-tracker) provides information about capacity of cement plants worldwide, technology used by each plant (OPC or blended), and future opening and closing dates, with partial information on emission reduction technology. Climate Trace (climatetrace.org) provides plant-level information on capacity, production and CO2 emissions.	Methodologies for computing emission factors for cement production depending on clinker content and the use of SCMs are described in the literature, see, e.g., [2, 3].
**Aluminum**	Climate Trace provides plant-level information on capacity, production and CO2 emissions. International Aliminium Institute (international-aluminium.org) provides data on aluminum production at monthly scale with country/region granularity.	International Aluminium Instite provides data on emission factors with breakdown by technological process.
**Pulp and paper**	Climate Trace provides plant-level information on capacity, production and CO2 emissions. The Spatial Finance Initiative Global Pulp and Paper Mill Database (sustainablefinance.ox.ac.uk/research/spatial-finance/geoasset-databases/pulp-and-paper-mill/) provides information on pulp and paper production facilities around the world. The database contains 3,403 facilities with information about location, operating status, plant type, product type, capacity, fuel, certification status and ownership where available.	The US Environmental Protection Agency provides emission factors for pulp and paper sector in the US (epa.gov/system/files/documents/2023-05/Pulp_and_Paper_Profile_RY2021_05-04-2023%20508.pdf).
**Power**	Global Integrated Power Tracker (globalenergymonitor.org/projects/global-integrated-power-tracker) and Global Power Plant Database (https://resourcewatch.org/data/explore/Powerwatch), provide plant-level capacity data; Climate Trace provides plant-level capacity, production and CO2 emissions data; Enerdata (commercial data provider) provides capacity and production data. Ember provides country-level production data.	Country level emission factors for various power generation technologies can be found in [4].

Supplementary Table 3: Plant-level and technology data availability for various sectors.

paper source category are combustion emissions. The decarbonization pathways for the pulp and paper sector are (a) moving away from fossil fuels as energy source (biomass or electrification) and (b) energy efficiency improvements such as using heat pumps (iea.org/energy-system/industry/paper).

Power sector

Decarbonization in the power sector primarily relies on switching to low-carbon generation sources such as renewables and nuclear, improving efficiency, and deploying carbon capture on fossil fuel plants. Data availability on power production capacity and emission factors is generally good. Emission factors for various fossil fuels are known and country-level differences are lower than for the steel sectors. The challenge is to accurately forecast electricity demand which is generally well predicted at short time scales but more difficult to forecast on longer time scales, where it depends on the evolutions of the society such as energy efficiency improvements, large-scale AI deployment etc.

4 Additional graphs and tables related to the projection of CO_2 emission factors for BF-BOF steel-making route.

Supplementary Figure 6 illustrates the statistical projection of BF-BOF emission factors based on the historical emission data from [6] for a sample of major steel producers.

Supplementary Table 4 lists the state of deployment of Best Available Technologies (BATs) for BF–BOF steelmaking decarbonization in major steel producing countries, their abatement potentials and the maximum abatement potentials for each country if technology deployment increases from the current level to 100%. Empty cells indicate that no specific information on country-wide adoption could be found for the specific technology. The following sources were used for technology adoption rates:

- Academic studies on technology adoption in China [7] and Germany [8].
- US Environmental Protection Agency white paper [9].
- World Steel in Figures (continuous casting data for all countries): worldsteel.org/data/world-steel-in-figures/world-steel-in-figures-2025).
- IEA Iron and Steel Technology Roadmap (worldwide and India adoption figures for CDQ and TRT: iea.org/reports/iron-and-steel-technology-roadmap.
- Steel Industry of Japan (jisf.or.jp/en/statistics/sij/index.html.
- Market reports on adoption of CDQ and PCI technologies: pmarketresearch.com/chemi/coke-dry-quenching-cdq-technology-market, globalgrowthinsights.com/market-reports/pulverized-coal-injection-pci-system-for-blast-furnaces-117781.
- Selected company reports.

Supplementary Table 4: State of deployment of Best Available Technologies (BATs) for BF–BOF steelmaking decarbonization in major steel producing countries, their abatement potentials and the maximum abatement potentials for each country if technology deployment increases from the current level to 100%. Empty cells indicate that no specific information on country-wide adoption could be found for the specific technology.

	**Technology**	Coke dry quenching (CDQ)	Coal moisture control (CMC)	Heat recovery from sintering and sinter cooler	Pulverized coal injection	Top-pressure recovery turbines (TRT)	Recovery of BF gas	Preheating of fuel and air for hot blast stove	Recovery of BOF gas and sensible heat	Flue gas waste heat recovery	Continuous casting	Recuperative or regenerative burner	Process control in hot strip mill	Waste heat recovery from cooling water	Heat recovery on annealing line	Automated monitoring
**Abatement potential ($kgCO_2/t$)**		50.05	16.32	73.83	60.41	27.30	5.84	26.03	48.19	19.24	36.35	46.50	25.61	2.95	30.06	24.04
**Country**	**Maximum abatement potential**	**State of technology deployment, per cent.**														
China	388.70	50	4.39		58.5	20	29.3				98.4					
India	405.47	30			45	40					93.9					
Japan	368.20	100			45	20	100				99					
United States	370.59	40			100	20					99.7					
Russia	377.33	50			90	20					84					
South Korea	399.35	50			45	20					98.2					
Germany	274.47	85		10	100	76		61			97.5	32	15			15

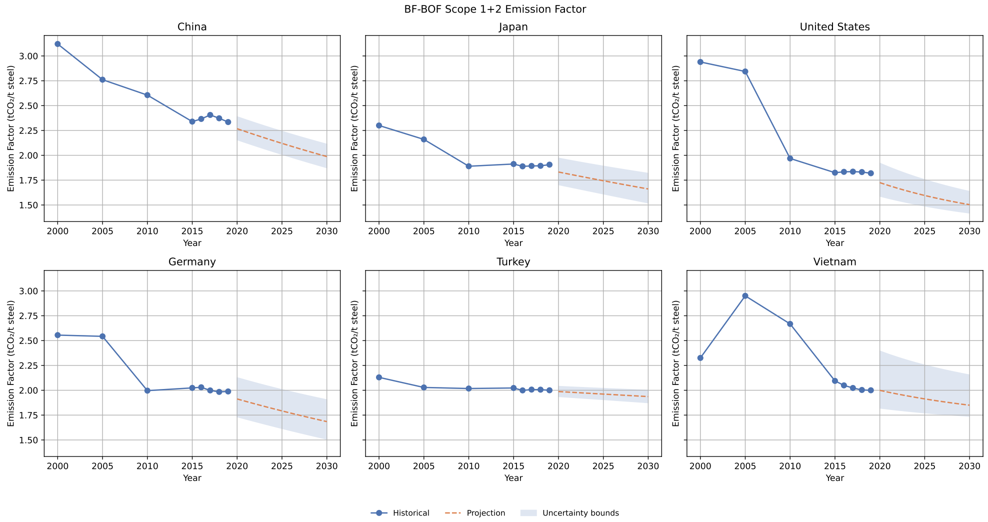

Supplementary Figure 6: Statistical projection of BF-BOF emission factor for a sample of countries based on historical data.

5 Availability of capacity and production data in GIST database

Supplementary Figure 7 illustrates the steel-making capacity coverage in the Global Iron and Steel Tracker database and Supplementary Figure 8 highlights the fraction of this capacity for which production data is available.

6 Sensitivity analysis of decarbonization scenarios of the electricity sector with respect to increased AI deployment

The evolving electricity consumption and procurement strategies of IT companies, particularly data centers can potentially alter the IEA projections and make these scenarios less relevant. We have therefore performed a sensitivity analysis to evaluate the potential impact of this increase of consumption on the carbon intensity of electricity.

To this end, we refer to the IEA's Energy and AI report (April 2025), which provides detailed projections of energy demand and supply for data centers through 2035. In its baseline scenario, global electricity use for data centers is expected to rise from 460 TWh in 2024 to around 1,000 TWh by 2030 (about 3% of global electricity generation). The largest increases are projected in mainland China (≈175 TWh, +170%), the United States (≈240 TWh, +130%), the European Union (≈45 TWh, +70%), and Japan (≈15 TWh, +80%). According to the report, about half of this additional demand will be met by renewable sources, with the remainder supplied by natural gas

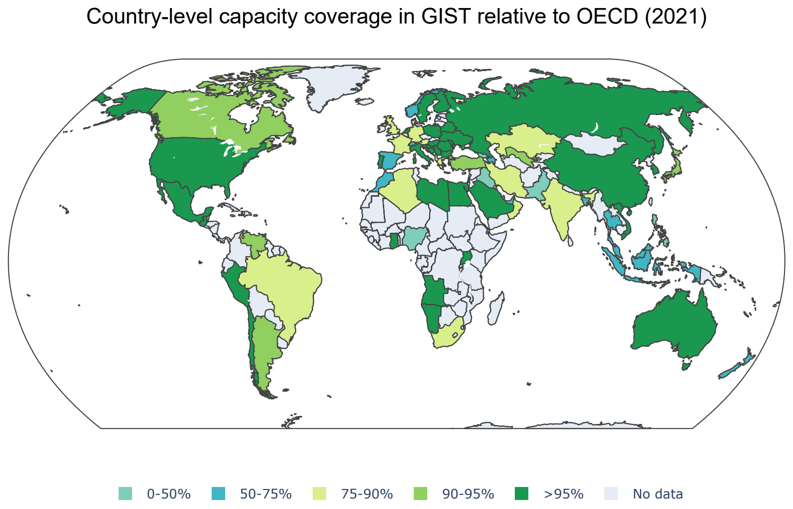

Supplementary Figure 7: Capacity coverage in GIST database. Production coverage in GIST database. The figure displays the ratio of total capacity listed in GIST database, to the total OECD capacity, for each country.

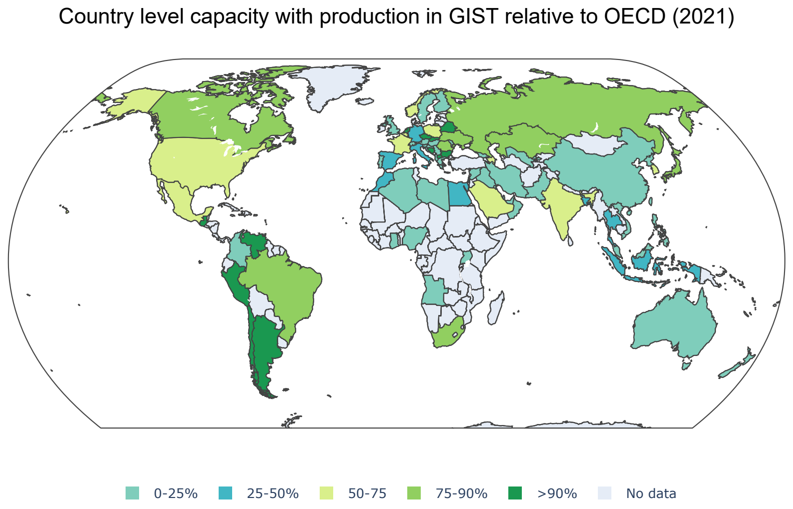

Supplementary Figure 8: Production coverage in GIST database. The figure displays the ratio of capacity for which production data is available in GIST database, to the total OECD capacity, for each country.

Scenario	Country	Baseline EF	AI-adjusted EF
IEA STEPS	United States	164.00	165.95
IEA APS	United States	107.00	111.45
IEA STEPS	China	385.00	382.36
IEA APS	China	342.00	339.94
IEA STEPS	Europe	99.00	100.00
IEA APS	Europe	70.00	71.21
IEA STEPS	Japan	252.00	251.34
IEA APS	Japan	230.00	229.66

Supplementary Table 5: Average emission factors for 2030 in IEA scenarios compared to emission factors adjusted for additional demand due to AI deployment.

(20%), coal (20%), and nuclear power (10%). A regional breakdown of the supply mix is not provided.

To evaluate the implications, we estimated the updated projected emission factors for electricity in 2030 in the IEA STEPS and APS scenarios (because in NZE scenario there is no regional breakdown of electricity generation), applying the above assumptions together with default technology-specific life cycle emission factors from the IPCC Fifth Assessment Report (Coal: 820 gCO2eq/KWh; Gas: 490 gCO2eq/KWh; Renewable (Solar): 45 gCO2eq/KWh; Nuclear: 12 gCO2eq/KWh). Supplementary Table 5 compares these updated factors with the original baseline scenario values. The results indicate that the impact of additional generation linked to AI is relatively small and does not materially alter the main estimates of our paper.

References

1. ClimeCo Corporation. *Low Carbon Cement Production Issue Paper* (2022). https://climateactionreserve.org/wp-content/uploads/2022/10/Low-Carbon-Cement-Issue-Paper-05-20-2022_final.pdf.

2. Shen, L. *et al.* Factory-level measurements on co2 emission factors of cement production in china. *Renewable and Sustainable Energy Reviews* **34**, 337–349 (2014).

3. Geng, Y., Wang, Z., Shen, L. & Zhao, J. Calculating of co2 emission factors for chinese cement production based on inorganic carbon and organic carbon. *Journal of Cleaner Production* **217**, 503–509 (2019).

4. Unnewehr, J. F., Weidlich, A., Gfüllner, L. & Schäfer, M. Open-data based carbon emission intensity signals for electricity generation in european countries–top down vs. bottom up approach. *Cleaner Energy Systems* **3**, 100018 (2022).

5. International Aluminum Institute. *Aluminium Sector Greenhouse Gas Pathways to 2050* (2021).

6. Zhang, J. *et al.* Iron and steel industry emissions: a global analysis of trends and drivers. *Environmental science & technology* **57**, 16477–16488 (2023).

7. An, R., Yu, B., Li, R. & Wei, Y.-M. Potential of energy savings and co2 emission reduction in china's iron and steel industry. *Applied energy* **226**, 862–880 (2018).

8. Arens, M., Worrell, E., Eichhammer, W., Hasanbeigi, A. & Zhang, Q. Pathways to a low-carbon iron and steel industry in the medium-term–the case of germany. *Journal of Cleaner Production* **163**, 84–98 (2017).

9. Jones, D. Available and emerging technologies for reducing greenhouse gas emissions from the iron and steel industry. *US EPA, Office of Air Quality Planning and Standards, Sector Policies and Programs Division* (2012).
